# Enhanced Levels of Chemokines and Their Receptors in the Colon of Microscopic Colitis Patients Indicate Mixed Immune Cell Recruitment

**DOI:** 10.1155/2015/132458

**Published:** 2015-04-09

**Authors:** Sezin Günaltay, Ashok Kumar Kumawat, Nils Nyhlin, Johan Bohr, Curt Tysk, Olof Hultgren, Elisabeth Hultgren Hörnquist

**Affiliations:** ^1^Örebro University, Department of Biomedicine, School of Health and Medical Sciences, 70182 Örebro, Sweden; ^2^University of Glasgow, Institute of Infection, Immunity and Inflammation, College of Medical, Veterinary and Life Sciences, Glasgow G128TA, UK; ^3^Örebro University, Division of Gastroenterology, Department of Medicine, Örebro University Hospital, School of Health and Medical Sciences, 70185 Örebro, Sweden; ^4^Örebro University Hospital, Department of Microbiology and Immunology, 70185 Örebro, Sweden

## Abstract

Microscopic colitis (MC), comprising collagenous colitis (CC) and lymphocytic colitis (LC), is a common cause of chronic diarrhea. Various immune cell infiltrations in the epithelium and lamina propria are seen in MC immunopathology. We compared gene and protein expressions of different immune cell attracting chemokines and their receptors in colon biopsies from MC patients in active disease or histopathological remission (CC/LC-HR) with controls, using qRT-PCR and Luminex, respectively. CC and LC patients with active disease demonstrated a mixed chemokine profile with significantly enhanced gene and/or protein expressions of the chemokines CCL2, CCL3, CCL4, CCL5, CCL7, CCL22, CXCL8, CXCL9, CXCL10, CXCL11, and CX_3_CL1 and the receptors CCR2, CCR3, CCR4, CXCR1, CXCR2, and CX_3_CR1. Enhanced chemokine/chemokine receptor gene and protein levels in LC-HR patients were similar to LC patients, whereas CC-HR patients demonstrated almost normalized levels. These findings expand the current understanding of the involvement of various immune cells in MC immunopathology and endorse chemokines as potential diagnostic markers as well as therapeutic candidates. Moreover, this study further supports the hypothesis that CC and LC are two different entities due to differences in their immunoregulatory responses.

## 1. Introduction

Microscopic colitis (MC), comprising collagenous colitis (CC) and lymphocytic colitis (LC), is characterized clinically by chronic watery diarrhea, abdominal pain, and/or weight loss. The diagnosis relies on typical histopathological features that are observed upon microscopic examination: lymphocytic infiltration of the epithelium and lamina propria as well as a damaged, flattened, and detached epithelial layer and in CC a characteristic thickened subepithelial collagen layer [1–4]. Biopsies from both LC and CC patients reveal a mixed inflammatory cell infiltrate in lamina propria, including T and B lymphocytes, plasma cells, eosinophils, neutrophils, mast cells, and macrophages [5–9]. Although the etiology of MC remains unclear, barrier dysfunction, increased numbers of immune cells, and/or immune response to luminal agents have all been suggested to be part of the pathogenesis [1].

**Figure 1:**
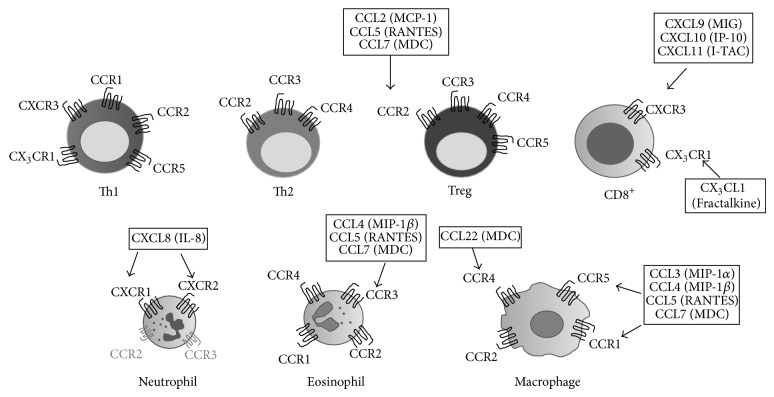
Summary of chemokines and their receptors investigated in this study. The chemokines interacting with their corresponding receptors are indicated once for each receptor but are valid for all cell types expressing these receptors.

Chemokines are small (~8–14 kDa) secreted proteins that orchestrate leukocyte migration by chemotaxis in homeostasis and inflammation [10]. Specificity in chemotaxis depends on both differential expressions of chemokines and their corresponding receptors expressed by leukocyte subsets [11]. Hence, dysregulated expression of chemokines and/or receptors may contribute to pathogenesis in different chronic inflammatory disorders [12]. Therefore, our aim in this study was to compare gene and protein expressions of a number of chemokines and their receptors, summarized in Figure 1, in colon biopsies from MC patients with active disease (CC/LC) or those clinically active but in histopathological remission (CC/LC-HR) with controls. To the best of our knowledge, this is the first comprehensive study demonstrating increased mucosal gene and protein expressions of immune cell attracting chemokines and their receptors in CC and LC. LC-HR patients showed similarities with LC patients in terms of enhanced chemokine and receptor expression levels, whereas CC-HR patients had normalized expressions. These results contribute to the knowledge of MC immunopathology, a subtler type of inflammatory bowel disease (IBD).

## 2. Materials and Methods

### 2.1. Patients

The MC patients underwent colonoscopy because of watery diarrhea, abdominal pain, and/or weight loss. Routine biopsy specimens were obtained from the proximal, transverse, and distal colon for confirmation of diagnosis through histopathological examination of paraffin embedded slides by an experienced gastropathologist. Histopathological criteria for CC were a diffusely distributed and thickened subepithelial collagen layer (≥10 *μ*m), epithelial damage such as flattening and detachment, inflammation in the lamina propria with mainly mononuclear cells, and increased numbers of intraepithelial lymphocytes (IELs). Histopathological criteria for LC were, in addition to epithelial damage and inflammation in the lamina propria, ≥20 IELs per 100 surface epithelial cells but with a normal collagen layer [1]. The clinical characteristics of the patients and controls were summarized in Table 1.

Four patients with an established diagnosis of CC and six patients with LC no longer fulfilled the histopathological criteria for MC despite clinical symptoms of the disease. These patients were therefore categorized as clinically active but histopathologically in remission (CC-HR/LC-HR) [13] and were analyzed separately (Table 1).

In gene and protein expression analyses, two patients with CC and two patients with LC were treated with budesonide at the time of colonoscopy, including only one patient having budesonide treatment 3 days before the colonoscopy. In protein expression analysis we included one more patient with CC, who was on budesonide treatment. These patients were identified in the graphs as circled symbols. We were unable to detect any effects of budesonide on the parameters tested.

Fourteen control individuals underwent colonoscopy due to changes in bowel habits (*n* = 2), iron deficiency anemia (*n* = 3), rectal bleeding (*n* = 2), follow-up after diverticulitis (*n* = 1), hemorrhoids (*n* = 1), irritable bowel syndrome (*n* = 1), colon cancer screening (*n* = 3), or abdominal pain (*n* = 1). The colonoscopy was macroscopically normal except for occasional diverticula in the left colon, and routine biopsy specimens from ascending, transverse, and distal colon revealed no pathological alterations.

Biopsy specimens for this study were obtained from the proximal colon with standard biopsy forceps and were immediately immersed in RNA*later* (Ambicon, Life Technologies, Foster City, CA, USA) and then stored at −80°C for later analysis.

### 2.2. RNA Isolation

Total RNA was isolated with miRNeasy Kits (Qiagen, GmbH, Hilden, Germany) according to the manufacturer's protocol and was quantified using a NanoDrop ND 1000 spectrophotometer (NanoDrop Technologies Inc., Wilmington, DE, USA).

### 2.3. Reverse Transcription and Quantitative Real Time Reverse Transcription Polymerase Chain Reaction (qRT-PCR)

All products used in the reverse transcription and qRT-PCRs were ordered from Applied Biosystems, Life Technologies (Austin, TX, USA). cDNA transcription of 500  ng/*µ*L total RNA was performed by High-Capacity cDNA Reverse Transcription Kits according to the manufacturer's protocol. The following TaqMan primer-probe sets were used: CCL2/MCP-1 (Hs00234140_m1), CCL3/MIP-1*α* (Hs00234142_m1), CCL4/MIP-1*β* (Hs99999148_m1), CCL5/RANTES (Hs00174575_m1), CCL7/MCP-3 (Hs00171147_m1), CCL22/MDC (Hs01574247_m1), CXCL8/IL-8 (Hs00174103), CXCL9/MIG (Hs00171065_m1), CXCL10/IP-10 (Hs01124251_g1), CXCL11/I-TAC (Hs04187682_g1), CX_3_CL1/Fractalkine (Hs00171086_m1), CCR1 (Hs00928897_s1), CCR2 (Hs00704702_s1), CCR3 (Hs00266213_s1), CCR4 (Hs00747615_s1), CCR5 (Hs99999149_s1), CXCR1 (Hs01921207_s1), CXCR2 (Hs01891184_s1), CXCR3 (Hs01847760_s1), and CX_3_CR1 (Hs01922583_s1). Normalization of qRT-PCR results was performed using the mean of three housekeeping genes GAPDH (Hs99999905_m1), GUSB (Hs99999908_m1), and 18S (Hs99999901_s1). For gene expression assays, TaqMan Fast Universal Master Mix was used with the thermal cycling parameters suggested in the manufacturer's protocol. The samples were run in the GeneBio-rad CFX96 Touch Real-Time PCR Detection System (Bio-rad Laboratories Inc., Hercules, CA, USA). Gene expressions were expressed relative to the average of the housekeeping genes. The comparative threshold cycle method was used to compare control and patient results [14].

### 2.4. Protein Extraction and Chemokine Analysis

The mean (±SEM) weight of biopsy specimens used for chemokine quantification was 5.6 ± 1.5 mg. The biopsies stored in RNA*later* were homogenized using Tissuelyser II (Qiagen, GmbH, Hilden, Germany) at 25 Hz for 5 times 1 minute in RIPA buffer (Sigma Aldrich, Steinheim, Germany) containing proteinase inhibitor cocktail (catalog number P8340, Sigma Aldrich). The homogenization mixture was centrifuged for 5 min at 10,000 rpm, and the supernatant was divided into aliquots and stored at −80°C until further processing. Tissue protein levels of CCL2, CCL3, CCL4, CCL7, CXCL8, CXCL10, and CX_3_CL1 were analyzed in duplicate by xMAP technology developed by Luminex (Austin, TX, USA). The concentrations were determined using the Milliplex Map Kit (catalog number SPR217) according to the manufacturer's instructions (Millipore, MA, USA). The levels of different chemokines from MC and controls were expressed as pg/mg tissue, according to a standard curve with known amounts of each analyte (Millipore).

### 2.5. Statistical Analysis

Data values were compared according to the nonparametric Mann-Whitney test with statistical significance set at *P* < 0.05 (GraphPad Prism 4, San Diego, CA, USA). Statistical outliers were any points found below first quartile (Q_1_) − 1.5 × interquartile range (IQR) and above Q_3_ + 1.5 × IQR. When present they are marked as crosses (X) in the graphs and excluded from the statistical analysis. The different patient groups including CC and LC and those in histopathological remission (CC-HR/LC-HR) were compared to noninflamed control tissues. Also, patients with active disease or with the same disease in histopathological remission were compared to each other.

## 3. Results

### 3.1. Increased Expressions of the Th1 and CD8^+^ T Cell-Associated Chemokines CXCL9, CXCL10, CXCL11, and CX_3_CL1 in MC Patients

**Figure 2:**
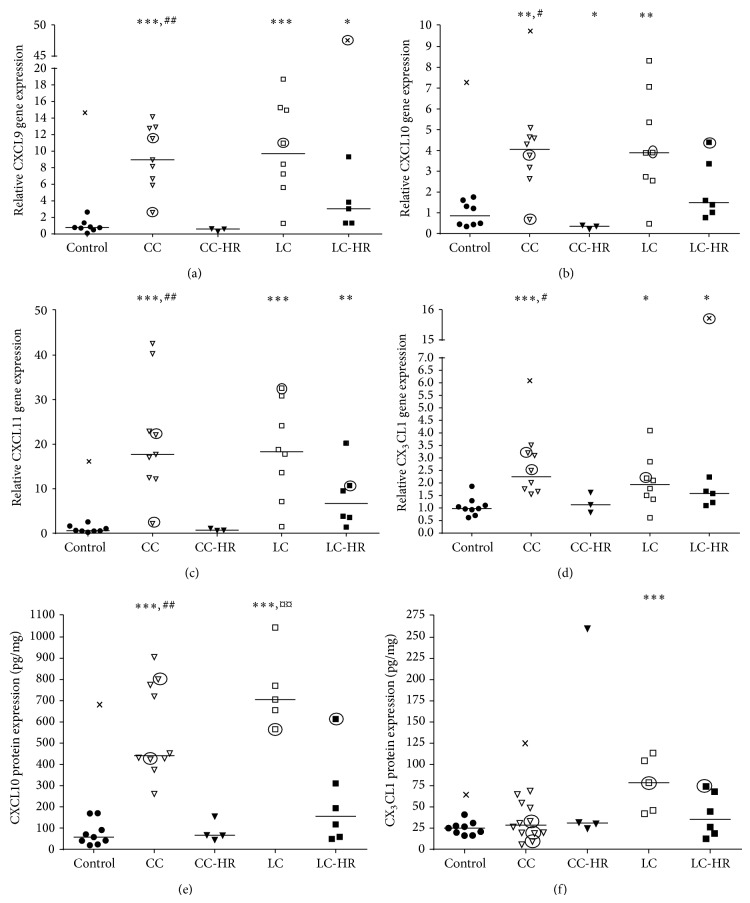
Gene and protein expressions of the Th1 and CD8^+^ T cell recruiting chemokines CXCL9, CXCL10, CXCL11, and CX_3_CL1. Each symbol represents one patient and the medians of the values are depicted as a line. Statistical outliers are marked as crosses (X) and budesonide treated patients are encircled. ^∗^ *P* < 0.05, ^∗∗^ *P* < 0.01, and ^∗∗∗^ *P* ≤ 0.001 versus controls, ^#^ *P* < 0.05, ^##^ *P* < 0.01 versus CC-HR, and ^¤^ *P* < 0.05, ^¤¤^ *P* < 0.01 versus LC-HR.

CXCL9, CXCL10, CXCL11, and CX_3_CL1 are important chemokines in Th1 and CD8^+^ T cell recruitment [15, 16]. Significantly increased gene expressions of CXCL9, CXCL10, CXCL11, and CX_3_CL1 were detected in CC patients compared to both controls and CC-HR patients (Figures 2(a)–2(d)). CC-HR patients also had decreased CXCL10 gene expression compared to controls (Figure 2(b)). Likewise, LC patients showed significantly enhanced CXCL9, CXCL10, CXCL11, and CX_3_CL1 gene expressions compared to controls (Figures 2(a)–2(d)). The enhanced gene expression levels of CXCL9, CXCL11, and CX_3_CL1 in LC-HR patients compared to controls indicate similarities with LC patients (Figures 2(a), 2(c), and 2(d)).

Protein expression of CXCL10 was enhanced in CC patients compared to controls and CC-HR patients (Figure 2(e)), in line with CXCL10 gene expression (Figure 2(b)). Likewise, LC patients had enhanced CXCL10 protein levels in comparison with both controls and LC-HR patients (Figure 2(e)). CX_3_CL1 protein level was significantly increased in LC patients only compared to controls (Figure 2(f)).

### 3.2. Gene Expression of CX_3_CR1 but not CXCR3 Was Upregulated in MC Patients

**Figure 3:**
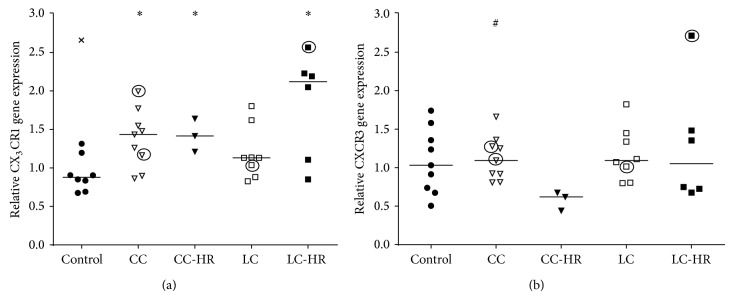
Relative gene expressions of the chemokine receptors CX_3_CR1 and CXCR3. Each symbol represents one patient, and the medians are depicted as a line. Statistical outliers are marked as crosses (X) and budesonide treated patients are encircled. ^∗^ *P* < 0.05 versus controls and ^#^ *P* < 0.05 versus CC-HR.

CXCL9, CXCL10, and CXCL11 bind to a common receptor, CXCR3 [15], whereas CX_3_CL1 only interacts with CX_3_CR1 [16]. CX_3_CR1 gene expression was significantly increased in CC, CC-HR, and LC-HR patients compared to controls (Figure 3(a)). The only significant alteration in CXCR3 gene expression was diminished expression in CC-HR patients compared to CC patients (Figure 3(b)) as well as a trend towards decreased expression compared to controls (*P* = 0.06, Figure 3(b)).

### 3.3. The Neutrophil Recruiting CXCL8 and Its Receptors CXCR1 and CXCR2 Showed Enhanced Gene and Protein Levels in MC Patients

**Figure 4:**
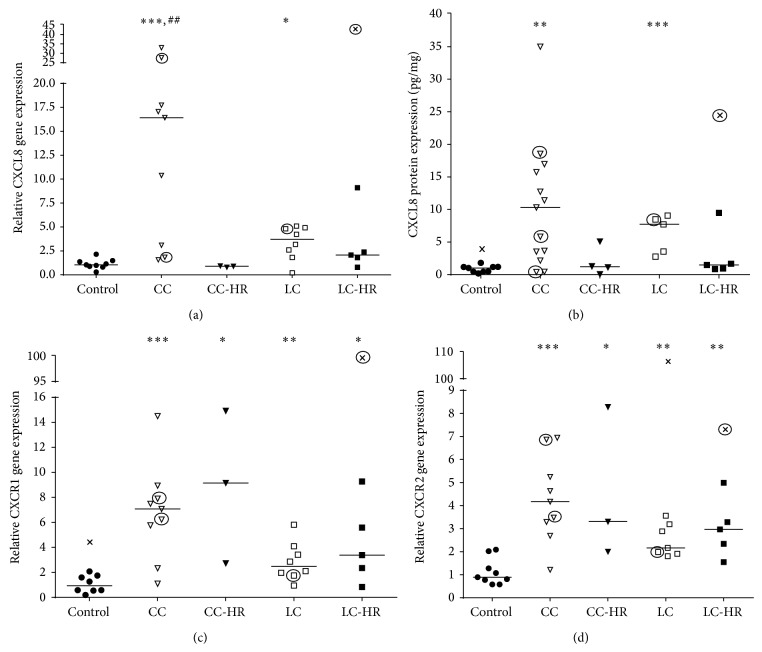
Relative gene and protein expressions of the neutrophil recruiting chemokine CXCL8, as well as gene expression of its receptors CXCR1 and CXCR2. Each symbol represents one patient and the medians of the values are depicted as a line. Statistical outliers are marked as crosses (X) and budesonide treated patients are encircled. ^∗^ *P* < 0.05, ^∗∗^ *P* < 0.01, and ^∗∗∗^ *P* ≤ 0.001 versus controls and ^#^ *P* < 0.05, ^##^ *P* < 0.01 versus CC-HR.

Gene and protein expressions of CXCL8 were significantly increased in both CC and LC patients compared to controls, with the highest expressions recorded in CC patients (Figures 4(a) and 4(b)). In addition, CC patients showed significantly increased CXCL8 gene expression compared to CC-HR patients (Figure 4(a)) as well as a trend towards increased protein levels (*P* = 0.06, Figure 4(b)). The two receptors, CXCR1 and CXCR2, showed significantly increased gene expressions in all MC patient groups compared to controls (Figures 4(c) and 4(d)).

### 3.4. Enhanced Gene and Protein Levels of CCL2, CCL3, CCL4, CCL7, and CCL22 in MC Patients

CCL2, CCL3, CCL4, CCL5, CCL7, and CCL22 are pleiotropic chemokines attracting Th1, Th2, regulatory T (Treg) cells, neutrophils, eosinophils, and/or macrophages [16–20].

**Figure 5:**
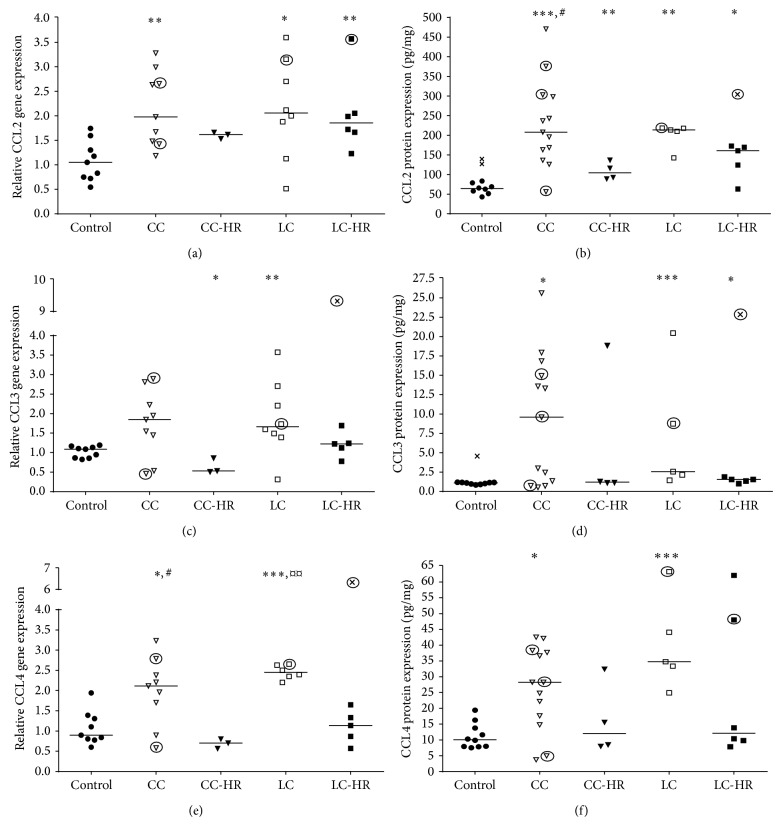
Relative gene and protein expressions of chemokines mediating recruitment of eosinophils, neutrophils, macrophages, Treg, Th1, and/or Th2 cells. Each symbol represents one patient and the medians are depicted as a line. Statistical outliers are marked as crosses (X) and budesonide treated patients are encircled. ^∗^ *P* < 0.05, ^∗∗^ *P* < 0.01, and ^∗∗∗^ *P* ≤ 0.001 versus controls, ^#^ *P* < 0.05 versus CC-HR, and ^¤^ *P* < 0.05, ^¤¤^ *P* < 0.01 versus LC-HR.

CCL2 showed increased gene as well as protein expression in CC, LC, and LC-HR patients compared to controls (Figures 5(a) and 5(b)). CC-HR patients showed intermediate levels of CCL2 protein, being significantly increased compared to controls but significantly decreased compared to CC patients (Figure 5(b)).

CCL3 showed a trend towards increased gene expression (*P* = 0.06, Figure 5(c)) and significantly increased protein levels (Figure 5(d)) in CC patients compared to controls. CC-HR patients demonstrated diminished CCL3 gene expression compared to controls (Figure 5(c)) but no change in protein levels (Figure 5(d)). Both gene and protein expressions of CCL3 in LC patients were increased compared to controls (Figures 5(c) and 5(d)). CCL3 protein levels were also enhanced in LC-HR patients compared to controls (Figure 5(d)).

CCL4 also showed enhanced gene and protein expressions in both CC and LC patients compared to controls (Figures 5(e) and 5(f)), whereas CC and LC patients in histopathological remission (CC/LC-HR) had normalized CCL4 gene and protein expressions (Figures 5(e) and 5(f)).

**Figure 6:**
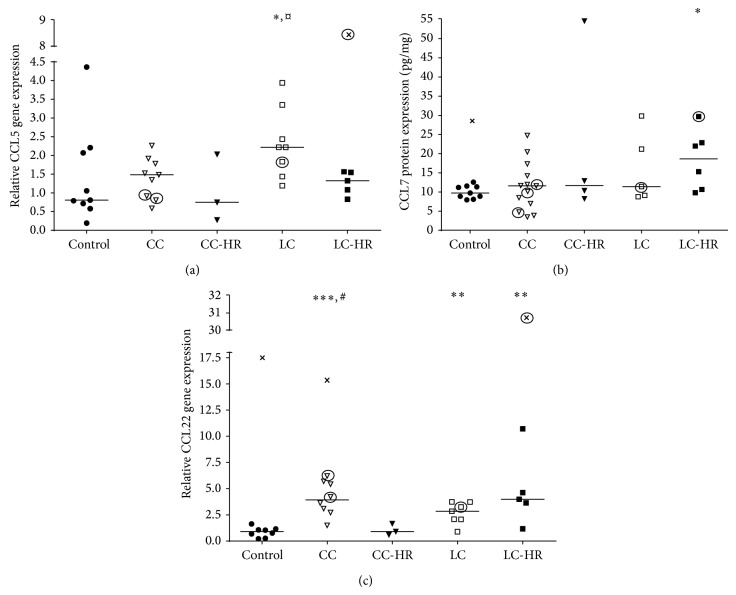
Relative gene and protein expressions of CC chemokines involved in recruitment of eosinophils, neutrophils, macrophages, Treg, Th1, and/or Th2 cells. Each symbol represents one patient and the medians are depicted as a line. Statistical outliers are marked as crosses (X) and budesonide treated patients are encircled. ^∗^ *P* < 0.05, ^∗∗^ *P* < 0.01, and ^∗∗∗^ *P* ≤ 0.001 versus controls, ^#^ *P* < 0.05 versus CC-HR, and ^¤^ *P* < 0.05 versus LC-HR.

Eosinophils, Th1, and Th2 cells are recruited by CCL5 [18–20], which was significantly increased in LC patients compared to both controls and LC-HR patients (Figure 6(a)).

CCL7 did not show significant changes in gene expression in any group of MC patients (data was not shown), whereas significantly increased protein expression was detected in LC-HR patients but not in any other patient group compared to controls (Figure 6(b)).

As opposed to CCL7, CCL22 gene expression was significantly increased in CC, LC, and LC-HR patients compared to controls (Figure 6(c)). In contrast, CC-HR patients had significantly decreased gene expression compared to CC patients, which was not different from the levels in controls (Figure 6(c)).

### 3.5. Increased Gene Expressions of Chemokine Receptors CCR2, CCR3, and CCR4 in MC

**Figure 7:**
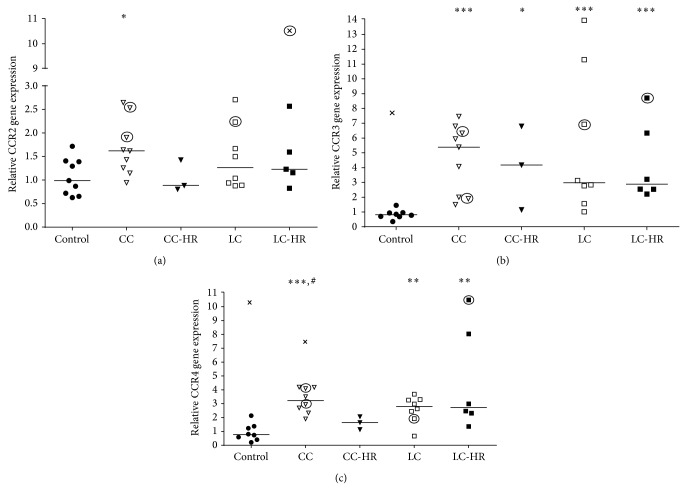
Relative gene expression of the chemokine receptors CCR2, CCR3, and CCR4. Each symbol represents one patient and the medians are depicted as a line. Statistical outliers are marked as crosses (X) and budesonide treated patients are encircled. ^∗^ *P* < 0.05, ^∗∗^ *P* < 0.01, and ^∗∗∗^ *P* ≤ 0.001 versus controls and ^#^ *P* < 0.05 versus CC-HR.

CCR2 binds CCL2, CCL5, and CCL7 and is expressed on neutrophils, eosinophils, macrophages, Th1, Th2, and Treg cells [21, 22]. It showed increased gene expression in CC patients only compared to controls (Figure 7(a)). In contrast, CCR3, binding CCL4, CCL5, and CCL7 and being expressed on neutrophils, eosinophils, Treg, Th1, and Th2 cells [18, 20, 23], had increased gene expression in all MC patient subgroups compared to controls (Figure 7(b)).

CCR4, binding to CCL22 and being found on macrophages, eosinophils, Th2, and Treg cells [18, 20], was significantly upregulated in CC, LC, and LC-HR patients compared to controls (Figure 7(c)). In CC patients CCL22 gene expression was also significantly upregulated compared to CC-HR patients (Figure 7(c)).

In contrast, neither CCR1 nor CCR5 showed any significant changes in gene expression (data was not shown).

## 4. Discussion

Although clinical and epidemiological data on MC are emerging the pathophysiology is still unclear and the searches for triggering factors and underlying dysfunctions in the mucosal immune system are still in an early stage [1–3]. Both CC and LC show infiltration primarily of T cells but also plasma cells, eosinophils, mast cells, macrophages, and neutrophils [5–8], which may be recruited by different chemokines and their receptors. The chemokines investigated in this study are all secreted from different cell types: CCL2, CCL3, CCL4, CCL5, CCL7, CXCL8, CXCL9, CXCL10, CXCL11, and CX_3_CL1 are secreted by colon epithelial cells [11, 21, 24, 25], whereas CCL22 is expressed by macrophages, mast cells, and dendritic cells [26, 27]. In addition, these chemokines can also be secreted by immune cells in the lamina propria, for example, macrophages (CCL2, CCL5, CCL22, CXCL8, and CXCL10), mast cells (CCL2, CCL5, and CCL22), eosinophils (CCL2, CCL5, and CCL7), and neutrophils (CXCL8 and CXCL10) [18, 21, 26, 28–32]. The gene and/or protein expressions of all these chemokines have been demonstrated to be increased in Crohn's disease (CD) and ulcerative colitis (UC) patients [21, 24, 33, 34]. However, to the best of our knowledge, there is only one study analyzing chemokine expression in MC patients, and that study was limited to observations of CXCL9 and CXCL10 in three LC patients compared to controls [35]. Therefore, we focused on chemokines and their receptors possibly involved in immune cell infiltration in MC immunopathology in order to increase our understanding of the disease mechanism(s) and eventually reveal possible therapeutic candidates.

Enhanced CXCL9, CXCL10, CXCL11, CX_3_CL1, and CX_3_CR1 gene and protein levels are likely involved in the CD8^+^ T cell infiltration [16, 33, 36] in the intraepithelial compartment and lamina propria of both CC and LC patients previously reported by us [9, 13]. These chemokines are also associated with Th1 cell recruitment. However, we observed increased numbers of CD8^+^ T cells only in MC patients and decreased numbers of CD4^+^ T cells. Therefore, these chemokines are more likely involved in CD8^+^ T cell recruitment in MC. IFN-*γ* is inducing CXCL9, CXCL10, and CXCL11 expressions, and we previously reported upregulated IFN-*γ* mRNA levels in CC, LC, and LC-HR patients, with normal levels in CC-HR patients [37]. These results corroborate our present results demonstrating enhanced expression of these chemokines in CC, LC, and LC-HR patients with normalized levels in CC-HR patients.

Enhanced gene and protein expressions of CXCL8 and its receptors CXCR1 and CXCR2 in MC patients suggest an important role for neutrophils, previously observed in the lamina propria of MC patients [3, 16, 19]. The significant upregulation of CXCR1 and CXCR2 gene expressions also in CC-HR and LC-HR patients could be related to neutrophil recruitment due to ongoing contact with the gut microbiota, as these patients still have histopathological evidence of inflammation, although not fulfilling the criteria for CC/LC diagnosis (gastropathologist Agnes Hegedus, personal communication).

In IBD patients increased CCL2 expression has been correlated with disease activity, mainly in areas of epithelial cell damage [24]. Our findings of enhanced CCL2 gene and protein expressions in both active and histopathological remission patients may likewise correlate with epithelial cell damage in MC, similar to IBD immunopathology [3].

Although CCL3, CCL4, CCL5, CCL7, and CCL22 attract many different cell types such as eosinophils, neutrophils, macrophages, Treg, Th1, and/or Th2 cells [16, 18, 19], decreased numbers of CD4^+^ T cells detected by us in MC patients [9, 13], these chemokines are likely involved mainly in eosinophil, neutrophil, and macrophage recruitment. Eosinophil infiltration in CC patients has previously been demonstrated [7, 38, 39]. Because of high levels of these chemokines also in LC and LC-HR patients, eosinophils may be involved also in LC immunopathology.

Infiltration of inflammatory macrophages, with their high IL-23 production [40], has been suggested as part of IBD immunopathology [41–43]. The present data, together with our previously reported enhanced gene expression of IL-23, may also suggest an important role of macrophages in MC immunopathology [37]. Accordingly, CCR2, CCR3, and CCR4 are predominantly expressed on eosinophils and macrophages [16, 20, 44]. However, Treg cells, found in increased amounts in lamina propria of MC patients [45], may be another source of these chemokine receptors [16, 20].

A potential limitation of this study is the small cohort of MC patients collected. However, as the majority of parameters investigated show statistically significant changes, we believe that the small cohort is not an obstacle in this study. In addition, we chose to analyze patients with clinical symptoms but not fulfilling the histological criteria for CC/LC separately, increasing our knowledge about these subgroups. MC can only be diagnosed upon histopathological examination and patients with an MC diagnosis usually do not undergo repeated colonoscopies. Nevertheless, as there is still no cure for MC and the medications only relieve disease symptoms, studies like this one are necessary to increase the understanding of the immunopathogenesis and to find new avenues for treatment. This study also further supports the legitimacy of MC as a “model” to study the role of changes in immune regulation and basic pathophysiology of IBD, where MC patients may reveal important immunoregulatory mechanisms.

## 5. Conclusion

One of the diagnostic criteria of CC and LC is increased numbers of lymphocytes in both the epithelium and the lamina propria, but infiltration of additional immune cells such as neutrophils, eosinophils, and macrophages is also observed. We found enhanced mRNA and protein expressions of a mixed profile of chemokines and their receptors in CC and LC patients. Interestingly, LC-HR showed similarities with LC patients, whereas CC-HR patients had almost normalized expression patterns. The contrasting expression patterns in CC and LC patients in histopathological remission (CC/LC-HR) further support the hypothesis of CC and LC being different entities. These results contribute to the knowledge of MC immunopathology by suggesting important immunoregulatory roles of chemokines and their receptors involved in recruitment of CD8^+^ T cells, Treg cells, neutrophils, eosinophils, and macrophages. The parameters investigated in this study might be important possible future therapeutic targets to interfere with cell recruitments in MC patients.

## Figures and Tables

**Table 1:** Clinical characteristics of patients and controls.

	CC^a^		CC-HR^b^		LC^c^		LC-HR^d^		Control	
	mRNA	Protein	mRNA	Protein	mRNA	Protein	mRNA	Protein
Number of patients	9	13	3	4	8	5	6	6	9	10
Male/female	1/8	2/11	0/3	0/4	0/8	0/5	0/6	0/6	6/3	5/5
Age (y)	66.7^e^ (35–84)	62.5 (35–84)	55 (50–64)	57.3 (50–64)	69.1 (49–86)	74 (65–86)	61 (24–80)	57.8 (24–80)	61.1 (29–88)	53.4 (28–78)
Duration of disease (y)	6.7 (0–17)	6.9 (0–17)	5 (1–9)	6 (1–9)	2.4 (0–7)	1.8 (0–6)	1.2 (0–3)	2.2 (0–7)	n/a	n/a

^a^CC: specimens from collagenous colitis; ^b^CC-HR: specimens from clinically active CC patients in histopathological remission; ^c^LC: specimens from lymphocytic colitis; ^d^LC-HR: specimens from clinically active LC patients in histopathological remission. ^e^Data are shown as mean (range).
